# Trends in Healthcare-Associated Infections Prevalence and Risk Factors: Repeated Point Prevalence Survey in a Milan Tertiary Hospital (2022–2025)

**DOI:** 10.3390/antibiotics15070641

**Published:** 2026-06-27

**Authors:** Flavia Pennisi, Martino Alberto Godoy, Tommaso Camuffo, Sabrina Caruccio, Giusy D’Alterio, Rosella Nebbia, Carola Simone, Arjun Sarabhai Verma, Carlo Signorelli, Giovanni Rezza, Matteo Moro

**Affiliations:** 1 National PhD Program in One Health Approaches to Infectious Diseases and Life Science Research, Department of Public Health, Experimental and Forensic Medicine, University of Pavia, 27100 Pavia, Italy; 2 Faculty of Medicine, University Vita-Salute San Raffaele, 20132 Milan, Italy; godoy.martino@hsr.it (M.A.G.); camuffo.tommaso@hsr.it (T.C.); caruccio.sabrina@hsr.it (S.C.); dalterio.giusy@hsr.it (G.D.); nebbia.rosella@hsr.it (R.N.); simone.carola@hsr.it (C.S.); sarabhaiverma.arjun@hsr.it (A.S.V.); signorelli.carlo@hsr.it (C.S.); rezza.giovanni@hsr.it (G.R.); 3 Infection Control Committee, IRCCS San Raffaele Hospital, 20132, Milan, Italy; moro.matteo@hsr.it

**Keywords:** healthcare-associated infections, drug resistance, infection control, public health, infections, cross infection

## Abstract

**Background:** Healthcare-associated infections (HAIs) and antimicrobial resistance are major burdens in tertiary care hospitals. Repeated point prevalence surveys (PPSs) offer a pragmatic approach to monitor temporal changes and guide infection prevention. **Objectives:** Characterize healthcare-associated infections (HAI) prevalence trends, microbiological profiles, antimicrobial resistance (AMR) patterns, and risk factors to refine prevention strategies and hospital policy. **Methods:** Four annual cross-sectional PPSs were conducted between 2022 and 2025 using the standardized ECDC protocol. Data from all eligible inpatients present at 08:00 on survey days were collected through systematic medical record review. Multivariable logistic regression was used to identify factors independently associated with HAI, with additional sensitivity analyses evaluating invasive device burden and hospital ward type. **Results:** Across the surveys, 3314 patients were included. Overall HAI prevalence was 11.3%. Infections were most frequent in intensive care units (31.2%), followed by medical (14.6%) and surgical (14.2%) wards. Bloodstream infections (25.7%) and lower respiratory tract infections (19.8%) were the most common. Multivariable analysis identified invasive device exposure as the strongest predictor, with central venous and urinary catheters showing robust independent associations and a clear dose–response relationship according to the number of devices. Pathogens were predominantly Gram-positive cocci (40.5%) and Enterobacterales (30.8%), with *Klebsiella pneumoniae* being the most frequent isolate (13.0%). Notably, 57.6% of *K. pneumoniae* isolates were resistant to third-generation cephalosporins. All tested *Acinetobacter baumannii* isolates were resistant to carbapenems. **Conclusions:** This repeated PPS reveals a persistently high HAI burden, associated with invasive device exposure and resistant pathogens. Because of the repeated cross-sectional design, causal inference cannot be established. Hospital-wide device stewardship and integrated surveillance are essential for guiding targeted prevention measures, refining antimicrobial policies, and adapting local responses to evolving resistance profiles.

## 1. Introduction

Healthcare-associated infections (HAIs) represent a major public health burden worldwide, particularly in tertiary care hospitals where complex case-mix, invasive devices, and frequent procedures increase infection risk [1,2,3]. HAIs contribute to prolonged hospital stays, increased healthcare costs, and adverse clinical outcomes [4,5].

To monitor and compare HAI burden across healthcare systems, the European Centre for Disease Prevention and Control (ECDC) coordinates standardized point prevalence surveys (PPSs) in acute care hospitals across the European Union (EU)/European Economic Area (EEA) [6,7]. These surveys provide harmonized estimates of HAI prevalence and enable benchmarking between countries and institutions. The latest ECDC PPS conducted in 2022 reported an overall HAI prevalence of 7.1%, corresponding to an estimated 4.3 million affected patients annually in the EU/EEA [7]. In Italy, prevalence reached 9.8%, indicating a substantial national burden. The most frequently reported HAIs include respiratory tract infections, urinary tract infections, surgical site infections, and bloodstream infections (BSIs) [6,7].

Antimicrobial resistance (AMR) further amplifies the clinical and economic impact of HAIs. Resistant pathogens are associated with higher mortality, longer hospital stays, and limited therapeutic options. Globally, AMR was directly responsible for 1.27 million deaths in 2019 and associated with nearly 5 million deaths [8]. More recent global estimates confirm this escalating burden: in 2021, bacterial AMR was associated with an estimated 4.71 million deaths worldwide and was directly attributable to 1.14 million deaths, with projections of up to 39 million attributable deaths by 2050 in the absence of effective intervention [9]. In the EU/EEA, antimicrobial-resistant infections account for more than 35,000 deaths each year, and recent European surveillance has documented a rising incidence of bloodstream infections caused by carbapenem-resistant *Klebsiella pneumoniae*. Reflecting this threat, the 2024 WHO Bacterial Priority Pathogens List ranks carbapenem-resistant *K. pneumoniae* among the highest-priority pathogens for which new therapeutic options are urgently needed [10]. Integrating HAI surveillance with microbiological and resistance data is therefore essential to guide infection prevention and control (IPC) strategies [11].

Repeated PPSs using standardized methodology provide a pragmatic approach to monitor trends and support local IPC planning [12]. However, temporally comparable repeated cross-sectional data from Italian tertiary care hospitals remain limited, particularly regarding HAI prevalence, infection types, microbiological patterns, and resistance profiles.

This study investigates HAI prevalence and trends at a large tertiary care hospital in Milan using PPS data from 2022–2025, aiming to characterize the microbiological profile and AMR patterns of these infections, identify associated risk factors, and classify HAI types, thereby providing an evidence base to refine prevention strategies, inform hospital policy, and optimize resource allocation [12].

## 2. Results

### 2.1. Patient Characteristics

Four annual PPSs conducted between 2022 and 2025 included 3314 patients. Overall, 51.6% were male and 48.4% female. Age distribution was 22.9% < 45 years, 30.5% 46–65 years, 23.7% 66–75 years, and 22.9% ≥ 76 years (Table 1).

Patients with HAIs were older than those without HAIs, with a median age of 69 versus 63 years and mean ages of 65.3 ± 15.7 versus 57.9 ± 21.6 years (*p*-value < 0.001).

Most patients were hospitalized in surgical (30.8%) and medical wards (30.6%), followed by rehabilitation units (12.1%). Central venous catheters (CVCs) were present in 18.2% of patients, peripheral venous catheters (PVCs) in 62.8%, urinary catheters (UCs) in 25.5%, and invasive mechanical ventilation in 4.1%. PVC data were unavailable for 903 patients (27.2%), corresponding to the 2022 PPS wave, because this variable was not captured in that survey dataset.

Overall, 36.7% of patients had undergone surgery since admission. Of these, 27% had a National Healthcare Safety Network (NHSN)-classified procedure, and 9.7% had minimally invasive or non-NHSN surgery. According to the McCabe score, 5.9% had a rapidly fatal disease, 17.5% an ultimately fatal disease, and 76.7% non-fatal conditions.

Median time from admission to PPS was 8 (2–10) days. Patients with HAIs had longer presurvey stays than those without HAIs (median 18 vs. 7 days).

### 2.2. Distribution of HAIs

A total of 409 HAIs were identified in 374 of 3314 patients, corresponding to a prevalence of 11.3% and 12.3 infections per 100 patients.

According to Table 2, BSIs were the most frequent type (105, 25.7%; 3.17 per 100 patients), followed by pneumonia and other lower respiratory tract infections (81, 19.8%; 2.44 per 100 patients), urinary tract infections (52, 12.7%; 1.57 per 100 patients), surgical site infections (51, 12.5%; 1.54 per 100 patients), and gastrointestinal infections (51, 12.5%; 1.54 per 100 patients). Of BSIs, 10.5% were CVC-related and 2.7% were PVC-related; 11.2% of SSIs were deep incisional or organ-space infections.

Sepsis accounted for 35 cases (8.6%; 1.06 per 100 patients), while remaining infection categories each represented <3% of HAIs.

The prevalence of HAIs was highest in intensive care units (31.2%), followed by medical (14.6%) and surgical wards (14.2%).

Annual HAI prevalence varied across survey years: 9.6% in 2022, 14.7% in 2023, 10.2% in 2024, and 11.1% in 2025. A formal linear trend analysis using logistic regression with survey year as an ordered variable showed no evidence of a statistically significant linear trend over time (aOR per year 1.01, 95% CI 0.92–1.10, *p*-value = 0.869). However, when survey year was modeled as a categorical variable, annual prevalence differed significantly across years (global *p*-value = 0.0076), mainly due to higher HAI prevalence in 2023 compared with 2022 (aOR 1.62, 95% CI 1.20–2.19, *p*-value = 0.002).

### 2.3. Risk Factors for HAIs

In bivariate analyses comparing patients with and without HAIs, significant differences were observed across age categories, hospital area, invasive device use, surgery since admission, McCabe score, and length of hospital stay (*p*-value < 0.05).

**Figure 1:**
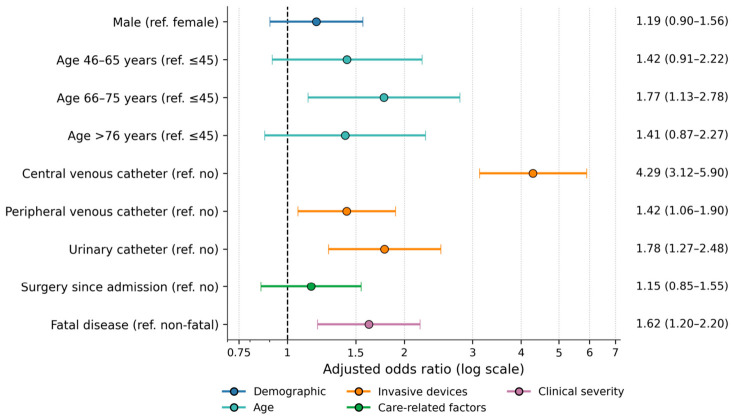
Multivariable analysis of factors associated with healthcare-associated infections. Adjusted odds ratios are shown with 95% confidence intervals on a logarithmic scale. HAI: healthcare-associated infections; ref: reference.

The primary multivariable logistic regression model is shown in Figure 1. CVC use was strongly associated with HAI (aOR 4.40, 95% CI 3.20–6.05, *p*-value < 0.001). UC use (aOR 1.78, 95% CI 1.28–2.48, *p*-value = 0.001) and PVC use (aOR 1.43, 95% CI 1.08–1.91, *p*-value = 0.014) were independently associated with HAI. Fatal underlying disease according to the dichotomized McCabe classification, including ultimately fatal and rapidly fatal disease, was also associated with higher odds of HAI in the primary model (aOR 1.58, 95% CI 1.17–2.14, *p*-value = 0.003). Patients aged 66–75 years had higher odds compared with those aged 0–45 years (aOR 1.77, 95% CI 1.13–2.78, *p*-value = 0.013).

Sensitivity analyses using the composite variable for the number of invasive devices (0, 1, ≥2) showed a dose–response relationship. Compared with patients without invasive devices, those with one device had higher odds of HAI (aOR 12.06, 95% CI 4.80–30.31, *p*-value < 0.001), and those with two or more devices had markedly higher odds (aOR 28.42, 95% CI 11.06–73.05, *p*-value < 0.001), and those with two or more devices had markedly higher odds (aOR 28.42, 95% CI 11.06–73.05, *p*-value < 0.001) (Table S1).

In the model excluding the McCabe score, associations with invasive device use remained similar (CVC: aOR 5.07, 95% CI 3.74–6.88; PVC: aOR 1.50, 95% CI 1.14–1.98), and age > 45 years was independently associated with HAI (Table S2). Effect estimates were consistent with the primary model. There was no statistical evidence that the association between invasive device burden and HAI differed according to surgical exposure (robust Wald test for interaction: χ^2^ = 2.37, df = 2, *p*-value = 0.305). The association between invasive device burden and HAI remained strong in the interaction model, while surgery since admission remained non-significant in the model without interaction (aOR 1.11, 95% CI 0.84–1.46, *p*-value = 0.456).

In a sensitivity model additionally adjusted for hospital ward type, the associations for CVC and urinary catheter use remained robust. CVC use was associated with higher odds of HAI (aOR 3.94, 95% CI 2.78–5.58, *p*-value < 0.001), as was urinary catheter use (aOR 1.89, 95% CI 1.35–2.63, *p*-value < 0.001). The association for PVC use was attenuated and no longer statistically significant after adjustment for hospital ward type (aOR 1.30, 95% CI 0.93–1.81, *p*-value = 0.128). Surgery since admission remained non-significant (aOR 1.31, 95% CI 0.91–1.88, *p*-value = 0.149), while fatal underlying disease remained associated with HAI (aOR 1.48, 95% CI 1.10–2.00, *p*-value = 0.009).

### 2.4. Pathogens Associated with HAIs

**Figure 2:**
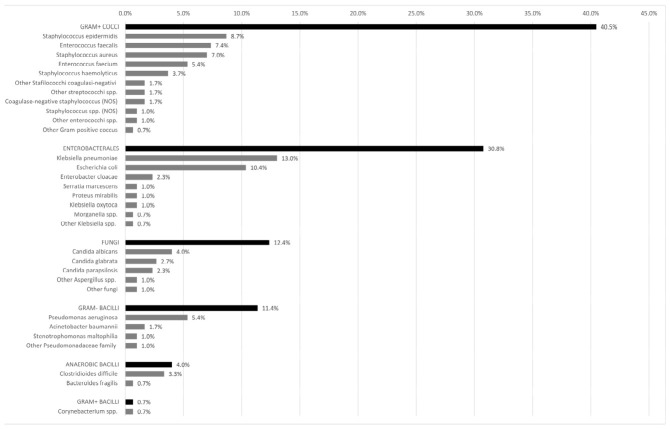
Frequencies and relative change in isolated pathogens by family and species. **Footnotes.** Percentages of individual species may not sum to the family total due to missing data. Only micro-organisms having at least 2 isolates have been reported. n: number. spp.: species pluralis. NOS: not otherwise specified.

Amongst the pathogens isolated, Gram-positive cocci accounted for 40.5%, Enterobacterales for 30.8%, fungi for 12.4%, and other Gram-negative bacilli for 11.4%. Anaerobic bacteria (4.0%) and Gram-positive bacilli (0.7%) represented smaller proportions (Figure 2).

The most frequently isolated Gram-positive cocci were *Staphylococcus epidermidis* (8.7%), *Enterococcus faecalis* (7.4%), and *Staphylococcus aureus* (7%). Among Enterobacterales, *Klebsiella pneumoniae* accounted for 13%, followed by *Escherichia coli* (10.4%) and *Enterobacter cloacae* (2.3%). Fungal isolates were primarily *Candida albicans* (4%), *Candida glabrata* (2.7%), and *Candida parapsilosis* (2.3%). Among other Gram-negative bacilli, *Pseudomonas aeruginosa* (5.4%), *Acinetobacter baumannii* (1.7%), and *Stenotrophomonas maltophilia* (1%) were identified.

In an exploratory-device-stratified descriptive analysis, 299 interpretable microbiological isolates were evaluated after excluding unavailable results, tests not performed, negative cultures, and unidentified microorganisms. Among isolates from HAI patients with a CVC in place, the most frequent pathogens were *Klebsiella pneumoniae* (n = 25, 15.1%), *Escherichia coli* (n = 16, 9.6%), *Enterococcus faecalis* (n = 12, 7.2%), *Enterococcus faecium* (n = 11, 6.6%), *Pseudomonas aeruginosa* (n = 11, 6.6%), *Staphylococcus epidermidis* (n = 10, 6.0%), and *Staphylococcus aureus* (n = 8, 4.8%). Among isolates from HAI patients with a urinary catheter in place, the most frequent pathogens were *Klebsiella pneumoniae* (n = 24, 13.7%), *Escherichia coli* (n = 16, 9.1%), *Enterococcus faecalis* (n = 14, 8.0%), *Staphylococcus aureus* (n = 13, 7.4%), *Staphylococcus epidermidis* (n = 12, 6.9%), *Candida albicans* (n = 8, 4.6%), and *Staphylococcus haemolyticus* (n = 7, 4.0%).

### 2.5. AMR Profiles of the Pathogens Causing HAIs

Antimicrobial susceptibility results for clinically relevant pathogen–antibiotic combinations are reported in Table 3. For Gram-positive pathogens, 28.6% of *Staphylococcus aureus* were resistant to oxacillin. Glycopeptide resistance was infrequent in *Enterococcus faecalis* (6.7% resistant), whereas *Enterococcus faecium* showed a higher prevalence (23.1% resistant), consistent with broader epidemiological patterns.

In Enterobacterales, resistance to third-generation cephalosporins was common. *Klebsiella pneumoniae* showed 57.6% resistance to third-generation cephalosporins and 18.2% to carbapenems. *Escherichia coli* exhibited 47.8% resistance to third-generation cephalosporins and 8.7% to carbapenems, while *Enterobacter cloacae* showed 28.6% and 14.3% resistance, respectively.

For non-fermenting Gram-negative bacilli, reduced carbapenem susceptibility was notable. *Pseudomonas aeruginosa* showed 23.1% resistance and 38.5% intermediate susceptibility to carbapenems, and all tested *Acinetobacter baumannii* isolates were carbapenem-resistant.

## 3. Discussion

### 3.1. Main Findings

This study, based on four consecutive PPSs conducted between 2022 and 2025, provides a repeated cross-sectional assessment of HAIs in a large tertiary care hospital using a standardized ECDC methodology. Overall, HAI prevalence showed annual variation, with a peak in 2023, but no evidence of a statistically significant linear trend across the four survey years. BSIs and pneumonia were the most frequent infection types overall. Although intensive care units had the highest concentration of cases, a substantial proportion of HAIs occurred in medical and surgical wards.

In multivariable analysis, invasive device exposure was the strongest independent determinant of HAI. CVCs and UCs were consistently associated with infection, and a dose–response relationship was observed with increasing numbers of devices. Age and underlying disease severity were also independently associated with HAI. The microbiological profile showed that Gram-positive pathogens were slightly more frequent than Gram-negative organisms, although *Klebsiella pneumoniae* was the most frequently isolated individual species, with substantial resistance to third-generation cephalosporins and carbapenems among selected species.

### 3.2. Interpretation of Findings

Annual HAI prevalence varied over the four-year study period, peaking in 2023, with no evidence of a statistically significant linear trend over time. This pattern suggests a persistent endemic burden rather than a sustained increase or decrease across survey years. The prevalence observed in our hospital remained higher than recent data reported by the Istituto Superiore di Sanità, which estimated an HAI prevalence of approximately 8% [13], as well as the adjusted European average of 6.2% reported in the latest ECDC survey [7]. Such differences are often attributed to variations in healthcare system organization, patient case-mix, and hospital structural complexity across regions [14,15]. Higher prevalence rates have been consistently reported in large referral centers managing patients with advanced diseases, frequent surgical interventions, and prolonged hospitalizations [16,17].

The predominance of BSIs and pneumonia reflects the high intensity of care in tertiary hospital settings [14] and substantial invasive device exposure [17]. Although intensive care units accounted for the largest proportion of cases, a considerable burden of HAIs was also observed in medical and surgical wards, indicating that infection risk is distributed hospital-wide rather than confined to critical care.

Invasive device exposure emerged as the strongest determinant of infection risk. The independent association observed for CVCs and UCs confirms their established epidemiological relevance [18]. The identification of PVCs as an independent risk factor, with a more modest effect size, is notable. Given their widespread use and perception as low-risk devices, even a moderate increase in risk may translate into a meaningful absolute burden at the population level [18,19]. The dose–response gradient suggests that cumulative device burden, rather than device type, drives infection risk. However, the very high odds observed for the composite invasive device burden variable should be interpreted cautiously. Device burden is likely to capture not only the biological and procedural risk associated with invasive devices, but also underlying illness severity, intensity of care, prolonged hospitalization, and clinical indication for device placement. Therefore, residual confounding by indication cannot be excluded, even after adjustment for measured demographic and clinical covariates. These findings should be interpreted as evidence of a strong association between device burden and HAI prevalence rather than as a direct causal estimate of device-attributable risk.

Although surgery is traditionally considered a major risk factor for HAIs [20], the association was no longer significant after multivariable adjustment, suggesting that the risk observed in unadjusted analyses may reflect confounding by correlated clinical exposures rather than an independent effect of the surgical procedure itself [21,22]. In tertiary care settings, surgical patients often have greater baseline severity and postoperative exposure to invasive devices, which may act as confounders or intermediates in the association between surgery and HAI. Adjustment for these factors attenuated the association between surgery and HAI, suggesting that the crude surgical risk may be partly explained by care-related exposures rather than by the procedure itself. In keeping with this, the additional interaction analysis did not show evidence that the association between invasive device burden and HAI differed by surgical exposure. This supports the interpretation that invasive devices partly account for the crude association between surgery and HAI, without indicating a differential device-related effect among surgical patients. These findings also highlight the need for cautious interpretation of adjusted estimates in cross-sectional surveillance studies, where care-related exposures may act both as confounders and as intermediates in the association between patient characteristics, procedures, and infection risk.

The distribution of isolated microorganisms showed a modest predominance of Gram-positive pathogens, while *Klebsiella pneumoniae* remained the single most frequently isolated organism, consistent with surveillance data from high-complexity hospitals and reflecting the increasing role of Enterobacterales in HAIs [7]. High resistance to third-generation cephalosporins suggests a substantial burden of extended-spectrum beta-lactamase-producing strains, while carbapenem resistance, though less frequent in Enterobacterales, remains clinically relevant.

Carbapenem resistance among non-fermenting Gram-negative bacilli, including universal resistance among tested *Acinetobacter baumannii* isolates, is particularly concerning given the limited therapeutic options [23,24]. These findings highlight the convergence of invasive device exposure, complex case-mix, and AMR in tertiary care settings. Although prescribing practices were not assessed, the resistance patterns underscore the need for coordinated IPC interventions and antimicrobial stewardship to reduce selective pressure and transmission of pathogens.

### 3.3. Implications for Infection Prevention and Control, Surveillance, and Stewardship

The findings reinforce invasive device stewardship as a central component of IPC in tertiary care settings. The strong independent associations observed for CVCs and UCs, together with the dose–response relationship according to device number, support strategies aimed at minimizing device utilization and exposure duration. Structured insertion bundles, standardized maintenance protocols, and daily reassessment of device necessity should be integrated into routine clinical governance [25].

The identification of PVCs as an independent risk factor highlights the cumulative impact of frequently used devices often perceived as low risk. Surveillance systems should capture not only high-risk devices but also high-volume exposures, enabling monitoring of dwell time, indication appropriateness, and adherence to maintenance standards.

A substantial portion of HAIs occurs outside intensive care units, underscoring the need for hospital-wide IPC measures supported by continuous surveillance [26]. Resistance patterns further call for integrated IPC and antimicrobial stewardship programs, linking device exposure, clinical risk factors, and susceptibility profiles to guide empiric therapy, containment strategies, and reduce selective pressure [27,28].

### 3.4. Strengths and Limitations

This study has limitations inherent to its repeated cross-sectional PPS design. PPSs do not permit estimation of incidence rates, temporal sequencing between exposure and outcome, or causal inference, and may preferentially capture infections of longer duration, potentially over-representing device-associated or severe infections while underestimating short-lived events.

The simultaneous assessment of exposures and outcomes is an intrinsic limitation of this study design and complicates the interpretation of adjusted associations, particularly where invasive device use and length of stay may act both as risk factors and consequences of infection. Data collection relied on structured medical record review. Despite adherence to standardized ECDC definitions and trained surveyors, misclassification and under-ascertainment remain possible. The complete-case approach in multivariable analyses may also have introduced selection bias if missingness was not random. In particular, PVC data were missing for the entire 2022 PPS wave, which may have reduced statistical power for PVC-specific analyses and may have biased PVC effect estimates if device use or infection risk differed between survey years. Accordingly, findings related to PVC exposure should be interpreted cautiously.

As a single-center study conducted in a tertiary care hospital, findings may not be fully generalizable, and clinical outcomes beyond the survey date were not captured.

Despite these limitations, several strengths are noteworthy. The consistent application of a harmonized ECDC protocol across four consecutive survey years ensured methodological comparability. Integration of clinical, device-level, and microbiological data enabled the assessment of epidemiological determinants alongside resistance patterns.

## 4. Materials and Methods

### 4.1. Study Design and Data Collection

This study was designed as a repeated cross-sectional analysis, based on annual PPSs [7] conducted between 2022 and 2025. The hospital infection prevention and control program is coordinated by a multidisciplinary infection-control team including infectious disease physicians, infection prevention and control nurses, microbiologists, antimicrobial stewardship specialists, and representatives from clinical departments. The team is responsible for HAI surveillance, implementation of infection prevention bundles, audit and feedback activities, staff training, outbreak investigation, and integration of clinical and microbiological data to guide local prevention strategies. During the study period, infection prevention activities included standard precautions, hand hygiene promotion, central-line insertion and maintenance bundles, urinary-catheter stewardship, surveillance of device-associated infections, antimicrobial stewardship activities, periodic audit and feedback, and staff education. These activities were implemented as part of routine hospital infection prevention and control practice and were not assigned or evaluated as experimental interventions within the PPS design. Therefore, the present study describes HAI prevalence and associated factors over time rather than estimating the causal effect of individual interventions.

Data collection followed the latest ECDC protocol [29] using a standardized case-finding methodology to enable cross-facility comparability. All wards providing acute, long-term, or rehabilitation care were eligible for inclusion, whereas day-care units, emergency departments without admitted patients, outpatient services, and non-clinical areas were excluded. Within participating wards, all patients physically present at 08:00 on the survey day and not discharged before data collection were included. Patients admitted after 08:00 were excluded. The survey target population comprised admitted inpatients only, in line with the ECDC PPS protocol. Outpatients, as well as day-care, day-surgery, and emergency-department patients who were not admitted, were not eligible; accompanying persons, visitors, and healthcare staff were likewise excluded.

Trained surveyors, including medical and nursing personnel from each participating ward, systematically reviewed individual medical records, medication charts, and microbiology reports to identify active HAIs and antimicrobial use. Data were recorded using the validated PPS form, recreated in Microsoft Excel worksheets, and captured demographic variables, clinical risk factors, device use, infection criteria, antimicrobial prescribing indications, and pressure ulcer information only in 2024–2025. No study-specific questionnaire was developed: the standardized, internationally validated ECDC PPS case-report form was applied without modification. Data were subsequently entered into a Microsoft Excel-based database, followed by protocol-mandated quality control procedures to ensure accuracy, completeness, and adherence to standardized case definitions. Preliminary data summarization and analysis were performed using the same informatics system. Organizational and technical phases were conducted and supervised by the PPS team of the Health Management Department of San Raffaele Hospital.

### 4.2. Outcome and Covariate Definitions

The primary outcome was the presence of at least one active HAI at the time of the survey, defined in accordance with standardized surveillance criteria used during the entire survey period. The outcome was analyzed as a binary variable (HAI present vs. no HAI).

For patients with confirmed HAIs, microbiological data were extracted from microbiology reports. All microorganisms isolated in association with HAIs were recorded. In case of polymicrobial infections, each isolate was analyzed separately. As an additional exploratory descriptive analysis, we examined the distribution of microbiological isolates according to the presence of major invasive devices, focusing on central venous catheters and urinary catheters. Because device exposure and microbiological findings were assessed cross-sectionally at the time of the PPS, these analyses were interpreted descriptively and were not used to infer device-attributable causality.

Pathogens were grouped into major taxonomic categories, and distribution was expressed as percentages of total isolates. Detailed site-specific reporting was limited to microorganisms accounting for more than 5% of isolates and represented by at least two isolates.

Antimicrobial susceptibility results were obtained from routine laboratory reports. For selected pathogen–antibiotic combinations of clinical relevance, resistance proportions were calculated as the number of resistant isolates divided by the total isolates tested for the specific antimicrobial agent. Resistance data were analyzed descriptively. Isolates were categorized as susceptible (S), intermediate (I), or resistant (R) according to international interpretive criteria based on clinician-reported data from the point of care.

Patient-level covariates included sex; age categorized as 0–45, 46–65, 66–75, and ≥76 years; year of survey; hospital area (medical, surgical, intensive care, pediatrics, obstetrics and gynecology, psychiatry, and rehabilitation); presence of invasive devices at the time of the survey (central venous catheter, peripheral venous catheter, urinary catheter, and endotracheal intubation); surgery since admission (any surgery vs. none in multivariable models); underlying disease severity assessed using the McCabe score (non-fatal, ultimately fatal, and rapidly fatal disease); and length of hospital stay calculated as days from admission to the survey date. A composite variable indicating the number of invasive devices in place (0, 1, ≥2) was constructed for sensitivity analyses.

### 4.3. Statistical Analysis

Descriptive statistics summarized patient characteristics overall and stratified by HAI status. Categorical variables were reported as frequencies and percentages, while continuous variables were summarized as medians and interquartile ranges.

Comparisons between patients with and without HAIs were performed using Pearson’s chi-square test or Fisher’s exact test for categorical variables and the Wilcoxon rank-sum test for continuous variables. Statistical significance was defined as a two-sided *p*-value < 0.05.

Multivariable logistic regression identified factors independently associated with HAIs. Covariates in the main model were selected *a priori* based on clinical relevance and comprised sex, age category, presence of invasive devices, surgery since admission, and McCabe score. Adjusted odds ratios (aOR) with 95% confidence intervals (CI) were estimated. Robust standard errors were applied, multicollinearity was assessed using the variance inflation factor, and a complete-case approach was adopted for multivariable modeling.

Hospital ward type was evaluated in an additional sensitivity model because it may capture differences in patient case-mix, care intensity, and device exposure across clinical areas. Length of stay was not included in the primary model to avoid overadjustment, given that in a point prevalence survey, it may represent both a risk factor for, and a consequence of, healthcare-associated infection.

Annual variation in HAI prevalence was assessed by survey year. A formal linear trend analysis was performed using logistic regression with survey year entered as an ordered continuous variable. Survey year was also evaluated as a categorical variable to assess differences in annual prevalence across survey years.

### 4.4. Sensitivity Analysis

Sensitivity analyses evaluated the robustness of the findings. These included re-estimation of the multivariable model under alternative specifications, including models incorporating the composite variable for the number of invasive devices (Table S1) and models excluding selected covariates to explore potential collinearity (Table S2). Effect estimates were compared across models to assess consistency in magnitude and statistical significance. As an additional sensitivity analysis, we tested whether the association between invasive device burden and HAI differed according to surgical exposure by adding an interaction term between surgery since admission and the composite invasive device variable (0, 1, or ≥2 devices) to the multivariable logistic regression model. The interaction was assessed using a robust Wald test.

## 5. Conclusions

This four-year repeated PPS demonstrates a persistently high burden of HAIs in a tertiary care hospital, with annual variation in prevalence but no evidence of a statistically significant linear trend over time. HAIs were predominantly represented by bloodstream infections and pneumonia. Invasive device exposure was the strongest independent determinant of infection, showing a clear dose–response relationship according to the number of devices. The microbiological profile was characterized by a slight predominance of Gram-positive pathogens, while substantial resistance to third-generation cephalosporins and carbapenems was observed among key Gram-negative species. These findings support prioritizing hospital-wide device stewardship strategies aimed at reducing unnecessary device exposure and duration, alongside integrated surveillance systems linking clinical and microbiological data. Continued standardized surveillance is essential to monitor annual variation over time and to evaluate the impact of infection prevention and control and antimicrobial stewardship interventions.

## Figures and Tables

**Table 1:** Demographic and clinical characteristics of the study population on survey date.

Characteristics	All Patients, n (% by Column)	Patients Without HAI, n (% by Row)	Patients with HAI, n (% by Row)	*p*-Value
Gender				
Male	1711 (51.6%)	1485 (86.8%)	226 (13.2%)	**<0.001**
Female	1603 (48.4%)	1455 (90.8%)	148 (9.2%)	
Age (years)				
0–45	744 (22.9%)	706 (94.9%)	38 (5.1%)	**<0.001**
46–65	994 (30.5%)	876 (88.1%)	118 (11.9%)	
66–75	772 (23.7%)	647 (83.8%)	125 (16.2%)	
>76	745 (22.9%)	656 (88.1%)	89 (11.9%)	
Years of survey				
2022	903 (27.2%)	816 (90.4%)	87 (9.6%)	0.007
2023	726 (21.9%)	619 (85.3%)	107 (14.7%)	
2024	822 (24.8%)	738 (89.8%)	84 (10.2%)	
2025	863 (26.0%)	767 (88.9%)	96 (11.1%)	
Area				
Medical	1013 (30.6%)	865 (85.4%)	148 (14.6%)	**<0.001**
Surgical	1022 (30.8%)	877 (85.8%)	145 (14.2%)	
Rehabilitative	400 (12.1%)	374 (93.5%)	26 (6.5%)	
Psychiatric	374 (11.3%)	372 (99.5%)	2 (0.5%)	
Pediatric	198 (6.0%)	191 (96.5%)	7 (3.5%)	
Obstetric and gynecological	169 (5.1%)	166 (98.2%)	3 (1.8%)	
Critical Care	138 (4.2%)	95 (68.8%)	43 (31.2%)	
CVC in place				
Yes	603 (18.2%)	417 (69.2%)	186 (30.8%)	**<0.001**
No	2710 (81.8%)	2522 (93.1%)	188 (6.9%)	
Missing	0 (0%)	0 (0%)	0 (0%)	
PVC in place				
Yes	1515 (62.8%)	1322 (87.3%)	193 (12.7%)	0.099
No	896 (37.2%)	802 (89.5%)	94 (10.5%)	
Missing	903 (27.2%)	816 (90.4%)	87 (9.6%)	
UC in place				
Yes	844 (25.5%)	649 (76.9%)	195 (23.1%)	**<0.001**
No	2470 (74.5%)	2291 (92.8%)	179 (7.2%)	
Missing	0 (0%)	0 (0%)	0 (0%)	
Intubation in place				
Yes	135 (4.1%)	96 (71.1%)	39 (28.9%)	**<0.001**
No	3175 (95.9%)	2840 (89.4%)	335 (10.6%)	
Missing	0 (0%)	0 (0%)	0 (0%)	
Surgery since admission				
No	2094 (63.3%)	1911 (91.3%)	183 (8.7%)	**<0.001**
Yes, NHSN surgery	892 (27.0%)	736 (82.5%)	156 (17.5%)	
Yes, minimally invasive/non-NHSN surgery	321 (9.7%)	287 (89.4%)	34 (10.6%)	
Missing	7 (0.2%)	6 (85.7%)	1 (14.3%)	
McCabe score				
Non-fatal disease	2474 (77.0%)	2282 (92.2%)	192 (7.8%)	**<0.001**
Ultimately fatal disease	564 (17.0%)	449 (79.6%)	115 (20.4%)	
Rapidly fatal disease	189 (5.9%)	131 (69.3%)	58 (30.7%)	
Missing	87 (2.6%)	78 (89.7%)	9 (10.3%)	
Days from admission to survey, median (IQR)	8 (4–17)	7 (3–15)	18 (10–34)	**<0.001**
Total	3314	2940	374	

**Footnotes.** Missing values are shown explicitly for variables with incomplete data to allow assessment of the complete-case approach used in multivariable modeling. PPS: point prevalence survey. HAI: healthcare-associated infection. n: number. CVC: central venous catheter. PVC: peripheral venous catheter. UC: urinary catheter. NHSN: National Healthcare Safety Network. IQR: interquartile range.

**Table 2:** Distribution of healthcare-associated infections by type, frequency, and predominant pathogens.

Type of Infection	HAIs, n (%)	HAIs per 100 Patients	Isolated Micro-Organisms, n (%) ^a^
BSI	105 (25.7%)	3.17	*Klebsiella pneumoniae, 15 (13.2%); Staphylococcus epidermidis, 15 (13.2%); Staphylococcus aureus, 10 (8.8%); Enterococcus faecalis, 8 (7.0%); Staphylococcus haemolyticus, 8 (7.0%); Enterococcus faecium, 6 (5.3%); Escherichia coli, 6 (5.3%)*
CVC infection	43 (10.5%)	1.30	
PVC infection	11 (2.7%)	0.33	
Other	51 (12.5%)	1.54	
PN and LRTI	81 (19.8%)	2.44	*Klebsiella pneumoniae, 11 (28.2%); Pseudomonas aeruginosa, 6 (15.4%); Aspergillus spp. (NOS), 3 (7.7%); Stenotrophomonas maltophilia, 2 (5.1%); Other Pseudomonadaceae family, 2 (5.1%); Enterobacter cloacae, 2 (5.1%); Acinetobacter baumannii, 2 (5.1%); Escherichia coli, 2 (5.1%)*
UTI	52 (12.7%)	1.57	*Escherichia coli, 12 (29.3%); Klebsiella pneumoniae, 7 (17.1%); Enterococcus faecalis, 4 (9.8%); Candida glabrata, 3 (7.3%); Proteus mirabilis, 3 (7.3%)*
SSI	51 (12.5%)	1.54	*Staphylococcus epidermidis, 7 (21.9%); Escherichia coli, 4 (12.5%); Staphylococcus aureus, 3 (9.4%); Enterococcus faecium, 3 (9.4%); Enterococcus faecalis, 2 (6.3%); Staphylococcus haemolyticus, 2 (6.3%); Candida albicans, 2 (6.3%); Pseudomonas aeruginosa, 2 (6.3%)*
Deep incisional or organ-space	46 (11.2%)	1.39	
Superficial incisional	3 (0.7%)	0.09	
Other	2 (0.5%)	0.06	
GI infection	51 (12.5%)	1.54	*Clostridioides difficile, 9 (20.0%); Escherichia coli, 6 (13.3%); Klebsiella pneumoniae, 4 (8.9%); Pseudomonas aeruginosa, 3 (6.7%); Enterobacter cloacae, 3 (6.7%); Staphylococcus epidermidis, 3 (6.7%)*
Sepsis	35 (8.6%)	1.06	*Klebsiella pneumoniae, 1 (25.0%); Escherichia coli, 1 (25.0%); Candida albicans, 1 (25.0%); Enterococcus faecalis, 1 (25.0%)*
Other HAI	26 (6.4%)	0.78	
Cardiac	9 (2.2%)	0.27	
Neural	8 (2.0%)	0.24	
Head and neck	6 (1.5%)	0.18	
Bone and joint	3 (0.7%)	0.09	
Skin and soft tissue infection	8 (2.0%)	0.24	*Pseudomonas aeruginosa, 1 (16.7%); Staphylococcus haemolyticus, 1 (16.7%); Staphylococcus aureus, 1 (16.7%); Clostridioides difficile, 1 (16.7%); Candida parapsilosis, 1 (16.7%); Other Klebsiella spp., 1 (16.7%)*
Total	409 (100%)	12.3	

**Footnotes.** HAI: healthcare-associated infections. n: number. BSI: bloodstream infection. CVC: central venous catheter. PVC: peripheral venous catheter. PN: pneumonia. LRTI: lower respiratory tract infection. UTI: urinary tract infection. SSI: surgical site infection. GI: gastrointestinal infection. spp.: species pluralis. NOS: not otherwise specified. ^a^ Only micro-organisms accounting for >5% and having at least 2 isolates have been reported, percentages are based on the total number of microorganisms isolated for each type of HAI.

**Table 3:** Antimicrobial susceptibility profiles of isolated pathogens. Values are expressed as number of isolates and percentage.

	OXA			GLY			C3G			CAR			PAN	
	S	I	R	S	I	R	S	I	R	S	I	R	Y	N
*Staphylococcus aureus*	15 (71.4%)	0(0%)	6 (28.6%)											
*Enterococcus faecalis*				14 (93.3%)	0(0%)	1 (6.7%)								
*Enterococcus faecium*				10 (76.9%)	0(0%)	3 (23.1%)								
*Other Enterococci*				1 (100%)	0(0%)	0(0%)								
*Other Enterococci* spp.				1 (50%)	0(0%)	1 (50%)								
*Enterobacter aerogenes*							1 (100%)	0(0%)	0(0%)	1 (100%)	0(0%)	0(0%)		
*Enterobacter cloacae*							5 (71.4%)	0(0%)	2 (28.6%)	6 (85.7%)	0(0%)	1 (14.3%)		
*Escherichia coli*							12 (52.2%)	0(0%)	11 (47.8%)	20 (87%)	1 (4.3%)	2 (8.7%)		
*Klebsiella pneumoniae*							12 (36.4%)	2 (6.1%)	19 (57.6%)	26 (78.8%)	1 (3.0%)	6 (18.2%)	1 (100%)	0(0%)
*Klebsiella oxytoca*							1 (50%)	0(0%)	1 (50%)	2 (100%)	0(0%)	0(0%)		
*Other Klebsiella* spp.							0(0%)	0(0%)	1 (100%)	0(0%)	0(0%)	1 (100%)		
*Morganella* spp.							1 (100%)	0(0%)	0(0%)	1 (100%)	0(0%)	0(0%)		
*Proteus mirabilis*							2 (100%)	0(0%)	0(0%)	2 (100%)	0(0%)	0(0%)		
*Serratia marcescens*							3 (100%)	0(0%)	0(0%)	3 (100%)	0(0%)	0(0%)		
*Acinetobacter baumannii*										0(0%)	0(0%)	5 (100%)		
*Pseudomonas aeruginosa*										5 (38.5%)	5 (38.5%)	3 (23.1%)		

**Footnotes.** OXA: oxacillin. GLY: glycopeptides. C3G: third-generation cephalosporins. CAR: carbapenems. PAN: pan-resistant. S: susceptible. I: intermediate. R: resistant. Y: yes. N: no. spp.: species pluralis.

## Data Availability

The data presented in this study are available on request from the corresponding author. The data are not publicly available due to privacy and ethical restrictions.

## Supplementary Materials

The following supporting information can be downloaded at: https://www.mdpi.com/article/10.3390/antibiotics15070641/s1, Table S1: Multivariable logistic regression analysis of risk factors for healthcare-associated infections, including number of devices, patient demographics, and clinical severity. Adjusted odds ratios (aOR) with 95% confidence intervals (CI) are reported. Table S2: Multivariable logistic regression model evaluating the association between vascular access devices and healthcare-associated infections, adjusted for patient demographics. Adjusted odds ratios (aOR) with 95% confidence intervals (CI) are reported.
