# Supplementary material for: Trends in Healthcare-Associated Infections Prevalence and Risk Factors: Repeated Point Prevalence Survey in a Milan Tertiary Hospital (2022–2025)
Source: Antibiotics (Basel). 2026 Jun 27;15(7):641. doi: 10.3390/antibiotics15070641 (PMC13405686; doi:10.3390/antibiotics15070641)
Supplement: Supplementary file 1 [file antibiotics-15-00641-s001.zip › antibiotics-4370335-supplementary.pdf]

Supplemental material

Supplemental Table S1. Multivariable logistic regression analysis of risk factors for healthcare-associated infections, including number of devices, patient demographics, and clinical severity. Adjusted odds ratios (aOR) with 95% confidence intervals (CI) are reported.

Variable	Adjusted OR (95% CI)	p-value
**Number of devices**		
1 (ref. 0)	12.06 (4.80-30.31)	<0.001
≥2 (ref. 0)	28.42 (11.06-73.05)	<0.001
**Gender**		
Male (ref. Female)	1.21 (0.92–1.58)	0.169
**Age (years)**		
46–65 (ref. 0–45)	1.39 (0.89–2.18)	0.149
66–75 (ref. 0–45)	1.52 (0.96–2.41)	0.073
>76 (ref. 0–45)	1.05 (0.65–1.70)	0.838
**Surgery since admission**		
Yes (ref. No)	1.13 (0.86–1.48)	0.389
**McCabe score***		
Fatal disease (ref. non-fatal disease)	1.53 (1.10–2.11)	0.011

*The McCabe score was dichotomised as non-fatal disease versus fatal disease. The fatal disease category includes both ultimately fatal and rapidly fatal diseases; rapidly fatal cases were not excluded from this model.

Supplemental Table S2: Multivariable logistic regression model evaluating the association between vascular access devices and healthcare-associated infections, adjusted for patient demographics.

Variable	Adjusted OR (95% CI)	p-value
**Gender**		
Male (ref. Female)	1.22 (0.93-1.60)	0.152
**Age (years)**		
46–65 (ref. 0–45)	1.357 (1.01–2.44)	0.047
66–75 (ref. 0–45)	1.99 (1.27-3.10)	0.003
>76 (ref. 0–45)	1.59 (1.01-2.51)	0.046
**Central catheter**		
Yes (ref. No)	5.07 (3.74–6.88)	<0.001
**Peripheral catheter**		
Yes (ref. No)	1.50 (1.14–1.98)	0.004

Adjusted odds ratios (aOR) with 95% confidence intervals (CI) are reported.
